# Predictors of anemia in preschool children: Biomarkers Reflecting Inflammation and Nutritional Determinants of Anemia (BRINDA) project

**DOI:** 10.3945/ajcn.116.142323

**Published:** 2017-06-14

**Authors:** Reina Engle-Stone, Grant J Aaron, Jin Huang, James P Wirth, Sorrel ML Namaste, Anne M Williams, Janet M Peerson, Fabian Rohner, Ravi Varadhan, O Yaw Addo, Victor Temple, Pura Rayco-Solon, Barbara Macdonald, Parminder S Suchdev

**Affiliations:** 1 Department of Nutrition, University of California, Davis, CA;; 2 Global Alliance for Improved Nutrition, Geneva, Switzerland;; 3 Division of Biostatistics and Bioinformatics, Department of Oncology, Johns Hopkins University, Baltimore, MD;; 4 GroundWork, Fläsch, Switzerland;; 5 Strengthening Partnerships, Results, and Innovations in Nutrition Globally, Arlington, VA;; 6 Department of Pediatrics, Emory University, Atlanta, GA;; 7 Department of Global Health, Rollins School of Public Health, Atlanta, GA;; 8 School of Medicine and Health Sciences, University of Papua New Guinea, Port Moresby, Papua New Guinea;; 9 Department of Nutrition for Health and Development, WHO, Geneva, Switzerland;; 10 Canadian Foodgrains Bank, Winnipeg, Manitoba, Canada; and; 11 Nutrition Branch, CDC, Atlanta, GA

**Keywords:** anemia, children, inflammation, iron, survey

## Abstract

**Background:** A lack of information on the etiology of anemia has hampered the design and monitoring of anemia-control efforts.

**Objective:** We aimed to evaluate predictors of anemia in preschool children (PSC) (age range: 6–59 mo) by country and infection-burden category.

**Design:** Cross-sectional data from 16 surveys (*n* = 29,293) from the Biomarkers Reflecting Inflammation and Nutritional Determinants of Anemia (BRINDA) project were analyzed separately and pooled by category of infection burden. We assessed relations between anemia (hemoglobin concentration <110 g/L) and severe anemia (hemoglobin concentration <70 g/L) and individual-level (age, anthropometric measures, micronutrient deficiencies, malaria, and inflammation) and household-level predictors; we also examined the proportion of anemia with concomitant iron deficiency (defined as an inflammation-adjusted ferritin concentration <12 μg/L). Countries were grouped into 4 categories on the basis of risk and burden of infectious disease, and a pooled multivariable logistic regression analysis was conducted for each group.

**Results:** Iron deficiency, malaria, breastfeeding, stunting, underweight, inflammation, low socioeconomic status, and poor sanitation were each associated with anemia in >50% of surveys. Associations between breastfeeding and anemia were attenuated by controlling for child age, which was negatively associated with anemia. The most consistent predictors of severe anemia were malaria, poor sanitation, and underweight. In multivariable pooled models, child age, iron deficiency, and stunting independently predicted anemia and severe anemia. Inflammation was generally associated with anemia in the high- and very high–infection groups but not in the low- and medium-infection groups. In PSC with anemia, 50%, 30%, 55%, and 58% of children had concomitant iron deficiency in low-, medium-, high-, and very high–infection categories, respectively.

**Conclusions:** Although causal inference is limited by cross-sectional survey data, results suggest anemia-control programs should address both iron deficiency and infections. The relative importance of factors that are associated with anemia varies by setting, and thus, country-specific data are needed to guide programs.

## INTRODUCTION

Preschool children (PSC) continue to have the highest burden of anemia compared with that of other population groups (estimated prevalence of 43% globally in 2011 and ≤71% in Central and West Africa) with only a 4-percentage point (pp) reduction from 1995 to 2011 (1). Furthermore, 2% of children globally (4.9% in Central and West Africa) were estimated to have severe anemia (1), which is associated with increased mortality (2). In a recent meta-analysis of data from African children, each 10-g/L increase in hemoglobin concentration was associated with a 24% mortality reduction (3). Iron-deficiency anemia (IDA) is associated with poorer cognitive and neurologic function with predicted long-term health and economic consequences (4) and is a major contributor to the estimated years lived with a disability in children and adolescents (5).

The etiology of anemia is complex and context-dependent and likely differs between mild and severe anemia (6). Various conceptual models have been developed to illustrate the pathways between proximal and distal determinants of anemia (4) including a framework that is specific to the Biomarkers Reflecting Inflammation and Nutritional Determinants of Anemia (BRINDA) project (7). Many potential causes of anemia are biologically interrelated, such as infections and nutritional status, and may also be driven by the same environmental and household characteristics, such as low socioeconomic status (SES), sanitation, and education (4). However, population-level assessments, particularly in low-resource settings, have frequently failed to adequately assess the etiology of anemia. Instead, risk factors for anemia have been inferred from limited indicators that are available from Demographic and Health Surveys or from small, nonrepresentative studies (8). This lack of information on the setting-specific risk factors for anemia is a major challenge to the development and evaluation of effective anemia-control programs and can result in an inefficient allocation of scarce public health resources.

Iron deficiency (ID) has been identified as an important cause of anemia for decades (9). As such, prevention and treatment strategies for populations at risk of anemia typically consist of approaches to improve iron status (10, 11). As these programs are scaled up, concerns have been raised about the potential adverse effects of providing additional iron to children, particularly to children who are iron replete, in settings with heavy burdens of infectious disease (12, 13).

In the absence of individual data on iron status, the prevalence of IDA (typically defined as having both ID and anemia) has been estimated by assuming that 50% of anemia is attributable to ID and, despite the rough nature of this estimate (14), the value has been widely referenced. Although the 50% estimate has been supported by some studies (8, 15), other studies have suggested that the proportion of anemia that is associated with ID may differ substantially in certain settings and subgroups (6, 16). In addition, estimates of the prevalence of ID and IDA depend on the indicator of iron status that is used and whether iron biomarkers are adjusted for inflammation (17–20).

The objective of this article was to assess the associations between anemia and posited risk factors for anemia in PSC in a variety of settings through a secondary analysis of a database of 16 population-based surveys that have been assembled under the BRINDA project (7, 21). We addressed the following research questions: *1*) What are the individual- and household-level factors that are associated with anemia and severe anemia in PSC, and how do these vary across settings with different burdens of infectious disease? *2*) What proportion of anemia is associated with ID, and how is the relation between anemia and ID affected by the application of new methods to adjust iron-status indicators for inflammation? and *3*) What is the relative strength of the association between anemia and factors that are independently associated with anemia?

## METHODS

We used data from the BRINDA project (www.BRINDA-nutrition.org); details of the project objectives and approach have previously been published (21). The BRINDA protocol was reviewed by the institutional review boards of NIH and was deemed to be non–human subjects research. Descriptions of the original surveys and BRINDA selection criteria are included in the methodologic summary in this issue (7). All 16 surveys (representing 14 countries) with PSC (age range: 6–59 mo) from the BRINDA project included measures of hemoglobin and inflammation [C-reactive protein (CRP) or α-1-acid glycoprotein (AGP)] and, thus, were eligible for the current analysis. However, we excluded Papua New Guinea from analyses that were related to iron status because only soluble transferrin receptor, and not ferritin, was measured.

### Laboratory analysis

Hemoglobin concentrations were measured at the time of blood collection with the use of a portable hemoglobinometer (HemoCue; all surveys except the United States) or after blood collection with a Beckman Coulter MAXM hematology flow cytometer (Beckman Coulter Inc.; United States only). Venous or capillary blood was collected from each respondent, and plasma or serum was stored at −20°C until analysis; one survey used dried blood spots. Ferritin, retinol-binding protein (RBP), CRP, and AGP concentrations were assessed with the use of a sandwich ELISA at the VitMin Laboratory in 9 of 16 PSC surveys (22). CRP was measured with the use of in-country methods in Colombia and Georgia (turbidimetry), Mexico (nephelometry), and the United States (immunoassay), and AGP was measured with the use of in-country methods in Nicaragua (turbidimetry) and Pakistan (immunoassay). Serum retinol was measured by HPLC in 5 surveys (Colombia, Nicaragua, Mexico 2006 and 2011, and Pakistan). Malaria was assessed with the use of microscopy in Kenya (2007 and 2010) and Côte d’Ivoire (23), the Paracheck Pf rapid diagnostic test (Orchid Biomedical System) in Liberia (24), and plasma histidine-rich protein 2 (Cellabs Pty Ltd.) in Cameroon (25). The methods for identifying data sets, inclusion and exclusion criteria, and data management for the BRINDA project have been described in the methodologic overview in this supplement, which is an open access publication (7).

### Categorization of countries by infection burden

Certain infectious diseases, such as malaria, contribute to anemia, but the burden of these diseases is not distributed evenly across countries. To understand how factors that are associated with anemia may differ for settings with varying burdens of infectious disease, we categorized countries into groups reflecting risk and burden of infectious disease on the basis of an examination of country-level characteristics, which is an approach that was adapted from Petry et al. (26). Specifically, each country was assigned scores that reflected sanitation, drinking water quality, and prevalences of malaria, diarrhea, and schistosomiasis (**Supplemental Table 1**). The scores were summed for each country and were used to divide countries into categories reflecting low, moderate, high, and very high burdens of infection. With the use of this approach, countries were grouped as follows: *1*) very high infection burden: Cameroon, Côte d’Ivoire, Kenya (2007 and 2010), and Liberia; *2*) high infection burden: Bangladesh, Laos, Papua New Guinea, and the Philippines; *3*) moderate infection burden: Colombia, Mexico (2006 and 2012), and Nicaragua; and *4*) low infection burden: Georgia and the United States.

Although alternate systems of defining the burden of infectious disease are possible, we adopted this system as a reasonable approach because of the following advantages: *1*) the categories were roughly consistent with geographic regions, which facilitated interpretation, and *2*) the 5 African data sets each represented a setting with a severe burden of malaria morbidity, and only these 5 data sets included individual-level information on malaria infection. The categories were also generally consistent with the prevalence of elevated CRP and AGP concentrations, although the categories were based on risk or burden of infection and not inflammation specifically.

### Definitions of outcomes and covariates

The primary outcomes were hemoglobin concentration (in grams per liter), anemia (defined as a hemoglobin concentration <110 g/L), and severe anemia (defined as a hemoglobin concentration <70 g/L) (27). Hemoglobin concentrations were adjusted for altitude when this information was available (*n* = 6 surveys) (27, 28). We applied the following categories to interpret the prevalence of anemia with regard to the public health burden—severe: >40%; moderate: 20.0–39.9%; mild: 5.0–19.9%; and normal: ≤4.9% (29).

A conceptual framework outlining the expected relations between anemia and selected potential risk factors for anemia, which is summarized in the introductory methods paper in this series (7), was used to guide the selection of covariates. Demographic characteristics included child age (expressed as a continuous variable, in months) and sex. Household SES was defined within each survey on the basis of household income in Papua New Guinea, the poverty-index ratio in the United States, and an asset score in all other surveys (except in Georgia, where income or assets were not measured). SES scores were dichotomized for bivariate analyses. Other household characteristics were as follows—location (urban or rural); sanitation conditions: improved or poor, comprising unimproved and open defecation (30); water source [improved or unimproved (30)]; and educational level of the head of household and/or the child’s mother (no education or primary education compared with secondary education or university). Child breastfeeding status was recorded as currently breastfeeding or breastfed in the past 24 h. Indicators of infection and inflammation included plasma or serum CRP and/or AGP (with inflammation defined as a CRP concentration >5 mg/L and/or AGP concentration >1 g/L, depending on the indicators available), and current or recent malaria (a positive diagnosis as assessed according to the laboratory methods previously described). Indicators of child nutritional status were as follows: stunting (height-for-age *z* score <−2 SDs), wasting (weight-for-height *z* score <−2 SDs), and underweight (weight-for-age *z* score <−2 SDs), which were calculated with the use of the WHO growth standards (31); ID [inflammation-adjusted ferritin concentration <12 μg/L (32, 33)]; and vitamin A deficiency (VAD) [inflammation-adjusted RBP or retinol concentration <0.70 μmol/L (34)].

To reduce confounding between indicators of ID and VAD with indicators of inflammation, regression-adjusted ferritin (33) and RBP or retinol concentrations were used to estimate individual iron and vitamin A status, respectively. Specifically, concentrations of each biomarker were adjusted for the presence of inflammation with the use of regression coefficients that were derived from linear regression models with the micronutrient biomarker as the outcome and CRP and/or AGP as continuous predictor variables; methods are described in detail in this issue (32, 34) and illustrated in **Supplemental Figure 1**. The adjustment method that was retained for this analysis used country-specific regression coefficients (internal regression coefficients) and reference CRP and AGP values of −2.26 ln(mg/L) and −0.52 ln(g/L), respectively. Other variables that were available for a small number of surveys and, thus, were not included in the current analyses were hemoglobinopathies, intestinal helminths, plasma or serum vitamin B-12, plasma or red blood cell folate, and plasma zinc.

### Statistical analyses

Descriptive analyses were conducted with SAS version 9.4 software (SAS Institute). The Taylor linearization method was used to obtain unbiased estimates that incorporated the sampling weight, strata, and cluster (as applicable). Sampling weights for each data set were provided by survey representatives. For pooled analyses, population-based weights were rescaled so that the sum of the weights within each study was proportional to the size of the population represented by the data set (e.g., all children within the selected age group in a country or region).

Associations between the primary outcomes (anemia and severe anemia) and each potential predictor were tested separately for each survey with the Rao-Scott chi-square test (except for age, which was examined as a continuous predictor variable with the use of a survey logistic regression procedure with Wald’s chi-square test). Pooled analyses were also conducted for each of the 4 infection groups. For bivariate analyses, prevalence ratios were estimated for each survey and infection group. Predictors of severe anemia were not examined in the countries in the low and medium infection burden groups because of the small number of cases.

We examined the relation between anemia and ID with and without adjustment of ferritin concentrations for inflammation with the use of the new, to our knowledge, regression-based method proposed by Namaste et al. (7), as previously noted. We calculated IDA as the proportion of all children with both anemia and ID. In addition, we calculated the proportion of anemic individuals with ID (IDA:all anemia; for comparison with the 50% estimate) and the prevalence of anemia in individuals with and without ID.

As an exploratory analysis, we developed a multivariable logistic regression model to identify independent predictors of anemia. Separate models were constructed for each of the 4 infection groups and for all anemia and severe anemia. The model development followed a block stepwise approach. Covariates were introduced into the model in blocks in the order of anticipated proximity in the causal pathway as follows: *1*) individual characteristics and proximal anemia determinants [age (continuous, in months), sex, ID, inflammation; and current or recent malaria (only countries with high infection burden)]; *2*) other variables related to nutritional status (breastfeeding, VAD, stunting, and wasting); *3*) household water and sanitation; and *4*) household demographics (rural or urban location, education of the head of the household or caregiver, and household SES). Within each geographic grouping, we limited the list of covariates to those available for all countries in that group with the exception of Papua New Guinea as previously noted.

The model construction proceeded as follows. Individual characteristics and proximal anemia determinants (group 1 variables, as described previously) as well as an indicator variable for the survey were forced into the model. Two-factor interactions of each predictor variable with age were introduced. Interactions with *P* > 0.10 were removed from the model in a stepwise manner. The second group of variables (group 2: other nutritional status variables) along with 2-factor interactions between each variable and age were introduced into the model. Nonsignificant variables (*P*-interactions > 0.10, *P*-main effects > 0.05) were removed in a stepwise manner, beginning with interactions (and retaining any main effects for which an interaction was significant). The same process was repeated for the third and fourth groups of variables. The final model was reviewed, and any nonsignificant terms were removed (except main effects for group 1 variables). Interactions of covariates with child age were described by examining the ORs for the covariate at different values for child age (12 and 48 mo). To assess heterogeneity by country in relations between anemia and each covariate, we constructed separate logistic regression models to examine the interaction (cross-product) between the survey indicator variable and each covariate of interest.

## RESULTS

### Population characteristics

Our study sample was restricted to participants with no missing values for hemoglobin and included a total of 29,293 PSC observations. Participants who were excluded because of missing hemoglobin values (*n* = 473; 1.6% of observations) did not differ from those who were included with regard to sex or SES except that children without hemoglobin measurements in the Mexico 2012 survey were more likely to have low SES than were children with hemoglobin measurements (data not shown). In addition, children without hemoglobin measurements were older in 6 surveys and younger in 5 surveys than were the children with hemoglobin measurements. All children were within the range of 6–59 mo of age, but the age range of participants differed by survey from 6–11 mo in Bangladesh to 12–59 mo in other surveys (**Table 1**). In pooled analyses, the mean child age was lower, and the proportion of children who were currently breastfeeding was greater in very high– and high-infection groups than in moderate- and low-infection groups (24.7 and 22.8 compared with 39.8 and 36.6 mo, respectively; 50.4% and 58.0% compared with 18.9% and 10.3%, respectively).

**TABLE 1:** Age, breastfeeding status, and anemia prevalence in preschool children by country and category of infection burden: the BRINDA project^1^

	*n*	Age,^2^ mo	Breastfeeding, %	Anemia, %	Severe anemia, %
Country					
Bangladesh	1491	8.3 (6.0–11.9)	NA	83.5 (81.1, 85.9)	1.1 (0.6, 1.6)
Cameroon	762	31.0 (12.0–59.9)	16.2 (13.0, 19.3)	54.3 (49.4, 59.3)	2.8 (1.4, 4.1)
Colombia	3864	37.6 (12.0–59.9)	NA	13.2 (11.7, 14.6)	0.1 (0, 0.3)
Côte d’Ivoire	742	31.6 (6.0–59)	NA	71.2 (66.4, 76.0)	7.3 (5.2, 9.4)
Georgia	2136	36.4 (12.0–60.0)	10.3 (8.5, 12.1)	23.0 (19.7, 26.3)	0.6 (0.1, 1.1)
Kenya 2007	873	20.0 (6.0–35.9)	56.3 (51.8, 60.7)	66.9 (63.0, 70.8)	3.2 (2.0, 4.4)
Kenya 2010	843	21.5 (6.0–35.9)	54.0 (50.0, 58.0)	71.5 (67.9, 75.1)	8.1 (6.1, 10.1)
Laos	479	33.1 (6.0–59.9)	31.5 (26.0, 36.9)	40.7 (31.2, 50.1)	0.5 (0, 1.2)
Liberia	1433	19.9 (6.0–35.9)	49.5 (46.3, 52.7)	59.5 (55.6, 63.4)	0.8 (0.3, 1.4)
Mexico 2006	1592	41.6 (12.0–59.9)	NA	20.7 (17.5, 23.8)	0.1 (0, 0.2)
Mexico 2012	2228	39.2 (12.0–59.9)	16.0 (12.8, 19.2)	16.8 (14.7, 19.0)	0.1 (0, 0.3)
Nicaragua	1423	33.4 (6.0–59.9)	60.9 (53.2, 68.6)	20.2 (15.7, 24.7)	0.2 (0, 0.5)
Pakistan	7477	27.3 (6.0–59.9)	76.0 (74.0, 78.0)	63.1 (61.8, 64.5)	4.4 (3.8, 5.0)
Papua New Guinea	871	31.4 (6.0–59.9)	42.6 (38.9, 46.3)	48.0 (42.3, 53.7)	2.4 (1.0, 3.7)
Philippines	1767	15.0 (6.0–23.9)	49.0 (45.6, 52.4)	41.9 (37.8, 45.9)	0.3 (0, 0.7)
United States	1312	36.6 (12.0–59.9)	NA	2.1 (1.0, 3.3)	0.0
Infection burden					
Low	3448	36.6 (12.0–59.9)	10.3 (8.5, 12.1)	2.4 (1.3, 3.5)	0 (0, 0)
Moderate	9107	39.8 (6.0–59.9)	18.9 (15.8, 21.9)	18.0 (16.4, 19.6)	0.1 (0, 0.2)
High	12,085	22.8 (6.0–59.9)	58.0 (56.0, 60.1)	56.9 (55.4, 58.3)	2.8 (2.4, 3.2)
Very high	4653	24.7 (6.0–59.9)	50.4 (47.9, 52.9)	68.1 (66.0, 70.1)	5.7 (4.8, 6.6)

1 All values are proportions (95% CIs), unless otherwise specified. Breastfeeding was reported as current breastfeeding or breastfeeding in the past 24 h. Anemia was defined as a hemoglobin concentration <110 g/L. Severe anemia was defined as a hemoglobin concentration <70 g/L. Countries were grouped as follows—low infection burden: Georgia and the United States; moderate infection burden: Colombia, Mexico (2006 and 2012), and Nicaragua; high infection burden: Bangladesh, Laos, Pakistan, Papua New Guinea, and the Philippines; and very high infection burden: Cameroon, Côte d’Ivoire, Kenya (2007 and 2010), and Liberia. BRINDA, Biomarkers Reflecting Inflammation and Nutritional Determinants of Anemia; NA, not available.

2 All values are means (minimums to maximums).

Anemia prevalence in PSC ranged from 2.1% in the United States to 83.5% in Bangladesh with weighted prevalence estimates of 2.4%, 18.0%, 56.9%, and 68.1% for low-, moderate-, high-, and very high–infection groups, respectively. Severe anemia affected 2.1–8.1% of PSC in the very high–infection group and 0.5–4.4% of PSC in surveys in the high-infection group but few PSC in the moderate- and low-infection groups. Anemia was characterized as a severe public health problem (>40%) in all surveys in the high- and very high–infection groups and as a mild (5.0–19.9%) or moderate (20.0–39.9%) public health problem in all other countries except the United States where the anemia prevalence was considered to be normal (≤4.9%).

Consistent with the infection-burden groupings, the prevalences of both ID and inflammation were lowest in the low-infection group (13.1% and 6.2%, respectively) and greatest in the group with a very high infection burden (54.4% and 63.0%, respectively) (**Table 2**). VAD prevalence appeared highest in the group with a high infection burden, but this was driven by the high prevalence of VAD in Pakistan (54.5%).

**TABLE 2:** Nutritional status, and prevalence of inflammation and malaria in preschool children by country and category of infection burden: the BRINDA project^1^

	Iron deficiency, %	Vitamin A deficiency, %	Inflammation, %	Malaria, %	Stunted, %	Wasted, %
Country						
Bangladesh	13.1 (10.5, 15.7)	8.2 (6.3, 10.2)	35.7 (32.2, 39.3)	NA	19.6 (15.8, 23.4)	17.8 (14.9, 20.7)
Cameroon	24.5 (20.8, 28.2)	13.7 (10.9, 16.5)	48.3 (43.0, 53.5)	25.8 (20.0, 31.5)	32.4 (28.0, 36.8)	3.8 (2.4, 5.2)
Colombia	11.1 (9.7, 12.4)	21.1 (19.3, 23.0)	18.8 (17.1, 20.5)	NA	14.0 (12.4, 15.5)	0.7 (0.3, 1.0)
Côte d'Ivoire	17.9 (14.9, 20.9)	11.3 (9.2, 13.4)	67.5 (63.7, 71.3)	27.3 (22.4, 32.2)	39.2 (34.3, 44.0)	13.8 (9.1, 18.5)
Georgia	0.3 (0.03, 0.5)	NA	24.7 (21.9, 27.6)	NA	11.9 (9.0, 14.9)	0.6 (0.2, 0.9)
Kenya 2007	59.1 (54.9, 63.3)	9.3 (6.8, 11.8)	65.5 (61.3, 69.7)	19.9 (16.1, 23.7)	25.3 (22.6, 28.1)	4.4 (2.6, 6.2)
Kenya 2010	39.3 (35.6, 42.9)	13.0 (10.5, 15.6)	61.8 (57.1, 66.5)	32.5 (28.4, 36.6)	26.1 (23.0, 29.2)	3.3 (1.8, 4.9)
Laos	23.0 (19.1, 26.9)	NA	43.9 (36.4, 51.4)	NA	50.8 (45.2, 56.4)	8.0 (4.7, 11.4)
Liberia	33.7 (30.1, 37.2)	12.1 (9.9, 14.4)	59.1 (55.6, 62.7)	29.4 (26.2, 32.6)	35.1 (31.9, 38.2)	9.2 (7.4, 10.9)
Mexico 2006	24.8 (21.6, 28.1)	NA	11.2 (9.0, 13.4)	NA	21.0 (17.1, 24.8)	1.3 (0.2, 2.3)
Mexico 2012	17.4 (15.2, 19.7)	14.6 (12.0, 17.1)	12.1 (9.6, 14.7)	NA	15.2 (13.0, 17.3)	0.8 (0.4, 1.3)
Nicaragua	47.9 (41.7, 54.2)	1.1 (0.5, 1.7)	24.0 (20.5, 27.5)	NA	18.9 (14.8, 23.1)	0.3 (0.04, 0.6)
Pakistan	51.6 (50.0, 53.1)	54.4 (52.2, 56.6)	35.5 (34.1, 37.0)	NA	44.3 (42.8, 45.7)	15.3 (14.4, 16.3)
Papua New Guinea	NA	13.7 (11.1, 16.4)	57.0 (52.5, 61.5)	NA	44.2 (38.9, 49.4)	4.3 (2.5, 6.1)
Philippines	35.8 (32.3, 39.2)	1.6 (0.4, 0.9)	26.0 (22.4, 29.5)	NA	26.4 (22.8, 30.1)	5.2 (3.6, 6.7)
United States	11.7 (8.9, 14.4)	NA	6.0 (4.5, 7.5)	NA	3.6 (1.9, 5.3)	0.5 (0, 1.1)
Infection burden						
Low	13.1 (10.2, 16.0)	NA	6.2 (4.8, 7.6)	NA	3.7 (2.1, 5.3)	0.5 (0, 1.0)
Moderate	23.3 (21.5, 25.1)	10.3 (9.1, 11.5)	12.9 (11.7, 14.1)	NA	17.6 (15.7, 19.5)	1.0 (0.5, 1.5)
High	43.8 (42.3, 45.3)	32.3 (30.8, 33.8)	33.3 (31.9, 34.7)	NA	37.6 (36.2, 39.0)	11.9 (11.1, 12.6)
Very high	54.4 (52.5, 56.4)	5.8 (5.0, 6.6)	63.0 (60.8, 65.2)	26.7 (24.5, 28.9)	30.2 (28.3, 32.1)	6.7 (5.3, 8.1)

1 All values are proportions (95% CIs). Iron deficiency was defined as an inflammation-adjusted ferritin concentration <12 μg/L. Vitamin A deficiency was defined as an inflammation-adjusted retinol concentration (Colombia, Mexico 2012, and Nicaragua) or a retinol-binding protein concentration (all other surveys) <0.70 μmol/L. Iron and vitamin A values were adjusted by regression with the use of reference CRP and AGP concentrations that were equivalent to the first decile of a reference population. Values were adjusted for CRP and AGP when both were available or for only CRP or AGP if only one was available. Inflammation was defined as a CRP concentration >5 mg/L or AGP concentration >1 g/L [only AGP data were available in Nicaragua and Pakistan, and only CRP data were available in Colombia, Georgia, Mexico (2006 and 2012), and the United States]. Stunted and wasted were defined as *z* scores <−2 SDs for height- or length-for-age and weight-for-height or -length, respectively, according to WHO growth standards. Countries were grouped as follows—low infection burden: Georgia and the United States; moderate infection burden: Colombia, Mexico (2006 and 2012), and Nicaragua; high infection burden: Bangladesh, Laos, Pakistan, Papua New Guinea, and the Philippines; and very high infection burden: Cameroon, Côte d’Ivoire, Kenya (2007 and 2010), and Liberia. AGP, α-1-acid glycoprotein; BRINDA, Biomarkers Reflecting Inflammation and Nutritional Determinants of Anemia; CRP, C-reactive protein; NA, not available.

### Bivariate associations with anemia and severe anemia

The factors that were associated with anemia differed by survey, but some patterns were apparent (**Tables 3**, **4**). Child age was positively associated (*P* < 0.05) with hemoglobin concentrations in all surveys except in Bangladesh, Liberia, and Kenya 2010, and older age was associated with a lower prevalence of anemia in all surveys except in Liberia and Bangladesh (data not shown). Other than child age, the most consistent factors that were associated with anemia across surveys were malaria (5 out of 5 surveys) and ID (12 out of 15 surveys). Current breastfeeding was associated with a greater prevalence of anemia in 8 of 11 surveys; in Pakistan, breastfeeding was associated with a lower prevalence of anemia. When child age (as a continuous variable) was included in the logistic regression model for anemia and breastfeeding, breastfeeding remained associated with anemia in Cameroon, Kenya 2007, Mexico 2012, and the Philippines and in the pooled analyses for moderate-, high-, and very high–infection groups.

**TABLE 3:** Bivariate associations between anemia and sex, breastfeeding, and nutritional status in preschool children by infection burden and by country: the BRINDA project^1^

	Sex, M	Breastfeeding	Stunting	Underweight	Wasted	Iron deficiency	Vitamin A deficiency
Very high infection burden	1.08 (1.03, 1.13)**	1.20 (1.14, 1.28)**	1.14 (1.08, 1.20)**	1.19 (1.14, 1.27)**	1.16 (1.09, 1.25) **	1.14 (1.08, 1.20)**	1.11 (1.02, 1.20)*
Cameroon	1.12 (0.98, 1.29)	1.50 (1.30, 1.72)**	1.31 (1.14, 1.51)**	1.40 (1.22, 1.61)**	1.62 (1.38, 1.89)**	1.39 (1.19, 1.62)**	1.29 (1.09, 1.54)**
Côte d'Ivoire	1.02 (0.94, 1.12)	NA	1.07 (0.95, 1.22)	1.14 (1.04, 1.25)**	1.08 (0.96, 1.21)	1.11 (1.00, 1.23)	1.06 (0.83, 1.37)
Kenya 2007	1.07 (0.97, 1.18)	1.17 (1.07, 1.29)**	1.19 (1.07, 1.31)**	1.25 (1.13, 1.37)**	1.15 (0.96, 1.37)	1.25 (1.11, 1.40)**	1.13 (0.95, 1.35)
Kenya 2010	1.12 (1.02, 1.24)*	1.14 (1.04, 1.25)**	1.12 (1.03, 1.22)**	1.20 (1.07, 1.33)**	1.31 (1.16, 1.49)*	1.07 (0.97, 1.18)	1.10 (0.96, 1.25)
Liberia	1.10 (1.00, 1.21)	1.08 (0.96, 1.21)	1.13 (1.02, 1.25)**	1.09 (0.98, 1.21)	1.03 (0.87, 1.20)	1.11 (1.00, 1.23)*	1.17 (0.98, 1.41)
High infection burden	1.01 (0.96, 1.06)	1.64 (1.49, 1.79)**	1.25 (1.20, 1.32)**	1.23 (1.19, 1.28)	1.16 (1.10, 1.23) **	1.54 (1.47, 1.64)**	1.15 (1.10, 1.20)**
Bangladesh	1.04 (1.01, 1.07)**	NA	1.04 (0.98, 1.11)	1.03 (0.97, 1.09)	0.98 (0.93, 1.04)	1.16 (1.11, 1.21)**	1.06 (0.96, 1.16)
Laos	1.21 (0.94, 1.56)	1.60 (1.33, 1.93)**	0.95 (0.74, 1.22)	1.38 (1.08, 1.78)*	1.73 (1.32, 2.26) **	1.80 (1.40, 2.33)**	NA
Philippines	0.96 (0.82, 1.14)	2.27 (1.90, 2.72)**	1.46 (1.26, 1.69)**	1.36 (1.20, 1.54)**	0.93 (0.63, 1.39)	1.95 (1.62, 2.36)**	1.64 (0.98, 2.77)
Pakistan	1.01 (0.98, 1.05)	0.93 (0.89, 0.98)**	1.19 (1.14, 1.24)**	1.14 (1.10, 1.18)**	1.08 (1.02, 1.13) **	1.45 (1.38, 1.51)**	0.98 (0.94, 1.02)
Papua New Guinea	0.99 (0.86, 1.15)	1.42 (1.21, 1.65)**	1.05 (0.87, 1.26)	1.39 (1.18, 1.64)**	1.82 (1.54, 2.15) **	NA	1.39 (1.16, 1.66)**
Medium infection burden	1.00 (0.83, 1.20)	1.59 (1.16, 2.17)**	1.64 (1.33, 2.00)**	1.35 (0.95, 1.92)	0.64 (0.28, 1.49)	1.45 (1.22, 1.72)**	1.41 (1.06, 1.85)*
Colombia	0.97 (0.79, 1.19)	NA	1.53 (1.18, 1.98)**	1.77 (1.14, 2.74)*	1.52 (0.67, 3.44)	1.52 (1.19, 1.94)**	1.34 (1.01, 1.78)*
Mexico 2006	0.84 (0.62, 1.14)	NA	1.53 (1.08, 2.15)**	1.35 (0.82, 2.21)	0.34 (0.07, 1.75)	1.33 (1.02, 1.74)*	NA
Mexico 2012	1.26 (0.95, 1.65)	1.66 (1.17, 2.36)**	1.79 (1.36, 2.35)**	1.06 (0.49, 2.29)	0.98 (0.33, 2.90)	1.42 (1.08, 1.88)*	1.59 (1.07, 2.36)*
Nicaragua	1.17 (0.86, 1.60)	1.11 (0.74, 1.68)	0.98 (0.64, 1.50)	1.43 (0.65, 3.13)	0.50 (0.10, 2.51)	2.09 (1.31, 3.33)**	2.46 (1.47, 4.12)**
Low infection burden	0.53 (0.25, 1.11)	1.67 (1.33, 2.04)**	0.48 (0.22, 1.00)*	0.55 (0.16, 1.89)	0.18 (0.03, 1.11)*	6.67 (2.63, 16.67)**	NA
Georgia	1.02 (0.83, 1.24)	1.66 (1.34, 2.06)**	1.13 (0.85, 1.49)	2.02 (1.17, 3.51)*	1.18 (0.36, 3.80)	0.84 (0.14, 5.15)	NA
United States	0.48 (0.20, 1.13)	NA	NA	NA	NA	8.64 (3.04, 24.57)**	NA

1 All values are prevalence ratios (95% CIs). Anemia was defined as a hemoglobin concentration <110 g/L. Breastfeeding was defined as currently breastfeeding or breastfed in the past 24 h. Controlling for child age (in months), the association was significant in Cameroon, Kenya 2007, Mexico 2012, and the Philippines and for pooled results for medium-, high-, and very high–infection groups. Stunting, underweight, and wasting were defined as *z* scores <−2 SDs for height-for-age, weight-for-age, and weight-for-height or -length, respectively, with the use of WHO growth standards. Iron deficiency was defined as an inflammation-adjusted ferritin concentration <12 μg/L (32). Vitamin A deficiency was defined as an inflammation-adjusted retinol concentration (Colombia, Mexico 2012, and Nicaragua) or a retinol-binding protein concentration (all other surveys) <0.70 μmol/L (34). Iron and vitamin A values were adjusted by regression with the use of reference CRP and AGP concentrations that were equivalent to the first decile of a reference population. Values were adjusted for CRP and AGP when both were available or for only CRP or AGP if only one was available. *^,^**Rao-Scott chi-square test: *P < 0.05, **P < 0.01. AGP, α-1-acid glycoprotein; BRINDA, Biomarkers Reflecting Inflammation and Nutritional Determinants of Anemia; CRP, C-reactive protein; NA, not available.

**TABLE 4:** Bivariate associations between anemia and inflammation, infections, and household characteristics in preschool children by infection burden and by country: the BRINDA project^1^

	Inflammation	Malaria	Reported diarrhea	Reported fever	Rural	Low SES	Poor sanitation	Unimproved water source	No or primary education
Very high infection burden	1.39 (1.32, 1.47)**	1.35 (1.28, 1.43)**	1.05 (1.00, 1.12)	1.12 (1.08, 1.16)**	1.14 (1.05, 1.23)**	1.08 (1.03, 1.14)**	0.91 (0.85, 0.97)**	0.96 (0.90, 1.03)	1.16 (1.07, 1.26)**
Cameroon	1.68 (1.42, 2.00)**	1.95 (1.70, 2.23)**	NA	NA	1.24 (1.01, 1.53)	1.54 (1.29, 1.83)**	1.21 (1.06, 1.37)**	1.22 (1.04, 1.43)*	1.65 (1.38, 1.96)**
Côte d'Ivoire	1.31 (1.16, 1.48)**	1.14 (1.01, 1.29)*	1.14 (1.01, 1.28)*	1.12 (1.04, 1.20)**	1.13 (0.99, 1.30)	1.16 (1.06, 1.28)**	1.10 (0.98, 1.24)	NA	1.00 (0.87, 1.16)
Kenya 2007	1.25 (1.11, 1.40)**	1.30 (1.19, 1.43)**	1.00 (0.89, 1.12)	1.06 (0.97, 1.15)	NA	0.93 (0.85, 1.01)	NA	0.95 (0.85, 1.05)	1.25 (1.05, 1.48)**
Kenya 2010	1.48 (1.34, 1.63)**	1.44 (1.34, 1.56)**	1.09 (1.00, 1.19)*	1.20 (1.11, 1.29)**	NA	1.08 (0.97, 1.21)	1.01 (0.68, 1.50)	1.05 (0.95, 1.15)	1.01 (0.89, 1.14)
Liberia	1.27 (1.15, 1.41)**	1.43 (1.31, 1.57)**	1.09 (0.99, 1.20)	1.16 (1.03, 1.31)**	1.10 (0.98, 1.24)	0.98 (0.86, 1.11)	1.00 (0.89, 1.13)	0.84 (0.70, 1.02)*	NA
High infection burden	1.19 (1.15, 1.25)**	NA	1.16 (1.10, 1.22)**	1.04 (0.95, 1.14)	0.89 (0.85, 0.94)**	0.97 (0.92, 1.03)	0.80 (0.76, 0.84)**	1.48 (1.34, 1.64)**	1.39 (1.29, 1.50)**
Bangladesh	1.08 (1.03, 1.14) **	NA	NA	NA	NA	NA	1.04 (0.98, 1.11)	0.98 (0.90, 1.08)	NA
Laos	1.07 (0.83, 1.39)	NA	1.01 (0.77, 1.34)	1.16 (0.86, 1.56)	1.94 (1.04, 3.62)**	1.11 (0.75, 1.64)	1.43 (0.97, 2.12)*	1.37 (0.96, 1.94)	1.25 (0.81, 1.92)
Philippines	1.29 (1.11, 1.50)**	NA	0.99 (0.79, 1.24)	1.34 (1.11, 1.62)**	0.99 (0.85, 1.14)	1.39 (1.08, 1.79)**	1.78 (1.45, 2.17)**	0.72 (0.60, 0.85)**	1.53 (1.30, 1.80)**
Pakistan	1.13 (1.09, 1.18)**	NA	1.14 (1.09, 1.18)**	1.14 (1.08, 1.21)**	0.97 (0.93, 1.02)	1.10 (1.06, 1.15)**	1.09 (1.02, 1.16)*	1.05 (0.95, 1.16)	1.08 (1.02, 1.14)**
Papua New Guinea	1.44 (1.24, 1.68)**	NA	NA	NA	1.24 (0.84, 1.82)	0.92 (0.69, 1.23)	1.42 (1.04, 1.95)*	1.23 (0.95, 1.59)	NA
Medium infection burden	1.08 (0.85, 1.35)	NA	1.85 (0.92, 3.70)	NA	1.22 (1.03, 1.46)*	1.59 (1.31, 1.94)**	0.67 (0.52, 0.86)**	1.02 (0.66, 1.56)	1.15 (0.87, 1.52)
Colombia	1.19 (0.92, 1.54)	NA	NA	NA	1.20 (0.96, 1.50)	1.60 (1.27, 2.02)**	0.86 (0.44, 1.66)	0.71 (0.35, 1.47)	0.82 (0.62, 1.09)
Mexico 2006	0.99 (0.66, 1.49)	NA	1.22 (0.46, 3.24)	NA	1.29 (0.95, 1.76)	1.77 (1.19, 2.63)**	1.51 (1.14, 2.02)**	NA	1.17 (0.84, 1.62)
Mexico 2012	1.16 (0.77, 1.75)	NA	3.69 (2.14, 6.39)**	NA	0.99 (0.76, 1.28)	1.33 (1.03, 1.72)*	NA	NA	NA
Nicaragua	1.57 (1.13, 2.18)*	NA	NA	NA	1.62 (1.10, 2.38)**	NA	1.57 (1.05, 2.35)**	1.35 (0.82, 2.22)	1.66 (1.22, 2.25)**
Low infection burden	1.52 (0.76, 2.94)	NA	0.91 (0.63, 1.32)	1.02 (0.78, 1.32)	1.04 (0.77, 1.40)	2.38 (1.16, 4.86)**	NA	NA	4.81 (1.49, 15.50)**
Georgia	1.12 (0.92, 1.37)*	NA	0.91 (0.63, 1.31)	1.02 (0.78, 1.32)	1.04 (0.77, 1.40)	NA	NA	NA	NA
United States	1.12 (0.42, 2.97)	NA	NA	NA	NA	2.38 (1.16, 4.86)*	NA	NA	4.81 (1.49, 15.50)

1 All values are prevalence ratios (95% CIs). Anemia was defined as a hemoglobin concentration <110 g/L. Any inflammation was defined as a CRP concentration >5 mg/L or AGP concentration >1 g/L [only AGP data were available in Nicaragua and Pakistan, and only CRP data were available in Colombia, Georgia, Mexico (2006 and 2012), and the United States]. Diarrhea was defined as reported symptoms in the past 24 h (Kenya 2007 and Kenya 2010) or 2 wk (Côte d’Ivoire, Georgia, Laos, Liberia, Mexico 2006, Mexico 2012, Pakistan, and the Philippines). Fever was defined as reported symptoms in the past 24 h (Kenya 2007, Kenya 2010, and Pakistan) or 2 wk (Côte d’Ivoire, Georgia, Laos, Liberia, and the Philippines). Poor sanitation was defined as unimproved sanitation and open defecation. The highest educational level was obtained for the caregiver of the child (Côte d’Ivoire, Cameroon, Kenya 2007, Kenya 2010, Laos, Nicaragua, Pakistan, and the Philippines) or head of the household (Colombia, Mexico 2006, and the United States). *^,^**Rao-Scott chi-square test: *P < 0.05, **P < 0.01. AGP, α-1-acid glycoprotein; BRINDA, Biomarkers Reflecting Inflammation and Nutritional Determinants of Anemia; CRP, C-reactive protein; NA, not available; SES, socioeconomic status.

Stunting, underweight, inflammation, low SES, and household sanitation were also associated with anemia in >50% of the surveys for which these variables were available, and VAD was associated with anemia in 5 of 12 surveys (Table 3). An elevated CRP concentration was associated with anemia in 8 of 14 surveys (0 of 5 surveys in low- and moderate-infection groups and 8 of 9 surveys in high- and very high–infection groups), whereas elevated AGP was associated with anemia in 10 of 11 surveys (data not shown). Any inflammation was associated with anemia in 9 of 10 surveys in the high- and very high–infection groups but in only 1 survey (Nicaragua) in the moderate- and low-infection groups (*n* = 6 surveys). Of the covariates examined, male sex (2 of 16 surveys) and the household water source (3 of 11 surveys) were associated with anemia in the smallest proportion of surveys.

The proportion of PSC with both anemia and any inflammation was 0.2%, 2.5%, 31.3%, and 47.9% in the countries with low, moderate, high, and very high infection burdens, respectively; in anemic children, the proportion with any inflammation was 9.1%, 13.7%, 37.4%, and 70.3%, respectively (**Supplemental Figure 2**).

Fewer predictors were identified for severe anemia (**Tables 5** **and** **6**); the most consistent predictors were malaria (4 of 5 surveys), poor sanitation (3 of 4 surveys), and underweight (6 of 9 surveys). Inflammation was associated with severe anemia in all 5 African countries but in none of the Asian countries. Male sex and recent diarrhea were not associated with severe anemia.

**TABLE 5:** Bivariate associations between severe anemia and sex, breastfeeding, and nutritional status in preschool children by infection burden and by country: the BRINDA project^1^

	Sex, M	Breastfeeding	Stunting	Underweight	Wasted	Iron deficiency	Vitamin A deficiency
Very high infection burden	1.07 (0.79, 1.45)	1.05 (0.69, 1.59)	2.13 (1.61, 2.78)**	2.44 (1.75, 3.45)**	2.38 (1.49, 3.70)**	0.72 (0.53, 0.97)*	2.33 (1.56, 3.57)**
Cameroon	1.21 (0.46, 3.19)	3.20 (1.36, 7.54)**	1.64 (0.85, 3.14)	3.39 (1.62, 7.06)**	7.77 (3.38, 17.88)**	0.61 (0.24, 1.58)	5.52 (2.04, 14.91)**
Côte d'Ivoire	0.76 (0.43, 1.33)	NA	2.50 (1.44, 4.34)**	1.93 (1.09, 3.40)*	1.60 (0.72, 3.59)	0.88 (0.48, 1.62)	2.09 (0.66, 6.60)
Kenya 2007	1.38 (0.71, 2.69)	1.04 (0.51, 2.09)	1.91 (0.92, 3.95)	4.20 (2.03, 8.68)**	4.93 (2.14, 11.36)**	0.56 (0.30, 1.07)	2.86 (1.06, 7.75)*
Kenya 2010	1.26 (0.79, 2.01)	0.88 (0.50, 1.56)	1.98 (1.31, 3.01)**	2.51 (1.40, 4.48)**	2.30 (0.98, 5.36)	0.81 (0.51, 1.29)	2.13 (1.24, 3.68)**
Liberia	2.38 (0.57, 9.85)	1.10 (0.29, 4.12)	2.55 (0.69, 9.49)	1.39 (0.32, 6.05)	1.18 (0.14, 9.69)	0.15 (0.04, 0.56)**	NA
High infection burden	1.03 (0.81, 1.32)	2.04 (1.41, 2.94)**	2.38 (1.82, 3.23)**	1.96 (1.52, 2.56)**	1.47 (1.09, 2.00)*	2.33 (1.79, 3.03)**	2.13 (1.64, 2.70)**
Bangladesh	1.32 (0.48, 3.61)	NA	4.70 (1.08, 20.42)*	2.51 (0.77, 8.17)	1.15 (0.33, 4.04)	36.49 (8.27, 161.01)**	1.13 (0.16, 8.13)
Laos	NA	NA	NA	NA	11.45 (0.58, 226.65)*	NA	NA
Philippines	3.08 (0.26, 36.90)	NA	2.69 (0.25, 29.31)	1.53 (0.13, 18.15)	NA	NA	NA
Pakistan	0.96 (0.75, 1.23)	0.85 (0.58, 1.23)	1.81 (1.35, 2.42)**	1.51 (1.16, 1.95)**	1.13 (0.83, 1.55)	1.58 (1.22, 2.05)**	1.05 (0.81, 1.37)
Papua New Guinea	1.70 (0.58, 4.94)	1.26 (0.37, 4.23)	6.18 (1.90, 20.14)**	5.37 (1.82, 15.85)**	4.40 (1.24, 15.65)*	NA	3.77 (1.56, 9.12)**

1 All values are prevalence ratios (95% CIs). Severe anemia was defined as a hemoglobin concentration <70 g/L. Breastfeeding was defined as currently breastfeeding or breastfed in the past 24 h. Controlling for child age (in months), the relation between severe anemia and breastfeeding was not significant in the high-infection group. Stunting, underweight, and wasting were defined as *z* scores <−2 SDs for height-for-age, weight-for-age, and weight-for-height or -length, respectively, with the use of WHO growth standards. Iron deficiency was defined as an inflammation-adjusted ferritin concentration <12 μg/L (32). Vitamin A deficiency was defined as an inflammation-adjusted retinol concentration (Colombia, Mexico 2012, and Nicaragua) or retinol-binding protein concentration (all other surveys) <0.70 μmol/L (34). Iron and vitamin A values were adjusted by regression with the use of reference CRP and AGP concentrations that were equivalent to the first decile of a reference population. Values were adjusted for CRP and AGP when both were available, or for only CRP or AGP if only one was available. *^,^**Rao-Scott chi-square test: *P < 0.05, **P < 0.01. AGP, α-1-acid glycoprotein; BRINDA, Biomarkers Reflecting Inflammation and Nutritional Determinants of Anemia; CRP, C-reactive protein; NA, not available.

**TABLE 6:** Bivariate associations between severe anemia and inflammation, infections, and household characteristics in preschool children by infection burden and by country: the BRINDA project^1^

	Inflammation	Malaria	Reported diarrhea	Reported fever	Rural	Low SES	Poor sanitation	Unimproved water source	No or primary education
Very high infection burden	6.25 (3.57, 10.00)**	2.78 (2.04, 3.85)**	1.15 (0.77, 1.69)	1.96 (1.45, 2.63)**	2.00 (1.23, 3.25)**	1.50 (1.11, 2.02)**	0.65 (0.45, 0.92)**	0.62 (0.42, 0.91)*	1.61 (0.88, 2.95)
Cameroon	5.60 (1.51, 20.82)**	8.64 (2.49, 30.01)**	NA	NA	1.30 (0.45, 3.74)	3.84 (1.36, 10.89)**	0.72 (0.28, 1.88)	3.92 (1.54, 9.95)**	15.45 (2.00, 119.56)**
Côte d'Ivoire	5.04 (1.97, 12.95)**	2.33 (1.40, 3.87)**	1.06 (0.52, 2.17)	2.41 (1.52, 3.83)**	2.94 (1.54, 5.61)**	2.78 (1.68, 4.60)**	2.25 (1.34, 3.76)**	NA	2.05 (0.62, 6.73)
Kenya 2007	3.16 (1.05, 9.46)*	1.70 (0.78, 3.68)	1.01 (0.46, 2.21)	1.27 (0.64, 2.53)	NA	1.17 (0.57, 2.41)	NA	1.62 (0.76, 3.49)	1.18 (0.36, 3.87)
Kenya 2010	9.89 (3.52, 27.80)**	2.89 (1.72, 4.87)**	1.50 (0.84, 2.67)	2.24 (1.33, 3.78)**	NA	0.94 (0.59, 1.49)	NA	1.33 (0.81, 2.21)	1.18 (0.50, 2.80)
Liberia	3.58 (0.91, 14.15)*	4.74 (1.14, 19.75)*	2.76 (0.67, 11.31)	NA	1.62 (0.48, 5.50)	2.67 (0.70, 10.16)	6.15 (1.28, 29.65)**	2.09 (0.51, 8.57)	NA
High infection burden	1.27 (0.97, 1.64)	NA	1.43 (1.10, 1.85)**	0.64 (0.40, 1.01)	1.07 (0.80, 1.42)	1.10 (0.84, 1.44)	0.72 (0.52, 0.99)*	2.28 (1.43, 3.63)**	2.90 (2.02, 4.15)**
Bangladesh	0.82 (0.31, 2.16)	NA	NA	NA	NA	NA	NA	7.95 (4.54, 13.93)**	NA
Laos	1.28 (0.07, 22.29)	NA	NA	4.49 (0.27, 73.28)	NA	0.64 (0.03, 11.74)	NA	NA	NA
Philippines	2.96 (0.26, 33.75)	NA	NA	2.07 (0.18, 23.43)	NA	NA	NA	NA	NA
Pakistan	1.07 (0.83, 1.39)	NA	1.25 (0.97, 1.61)	1.12 (0.76, 1.64)	1.46 (1.10, 1.94)**	1.79 (1.37, 2.33)**	2.06 (1.45, 2.91)**	1.23 (0.81, 1.88)	1.27 (0.90, 1.78)
Papua New Guinea	NA	NA	NA	NA	2.22 (0.47, 10.43)	2.40 (0.77, 7.53)	NA	2.72 (1.24, 5.95)**	NA

1 All values are prevalence ratios (95% CIs). Severe anemia was defined as a hemoglobin concentration <70 g/L. Any inflammation was defined as a CRP concentration >5 mg/L or AGP concentration >1 g/L [only AGP data were available in Nicaragua and Pakistan, and only CRP data were available in Colombia, Georgia, Mexico (2006 and 2012), and the United States]. Diarrhea was defined as reported symptoms in the past 24 h (Kenya 2007 and Kenya 2010) or 2 wk (Côte d’Ivoire, Georgia, Laos, Liberia, Mexico 2006, Mexico 2012, Pakistan, and the Philippines). Fever was defined as reported symptoms in the past 24 h (Kenya 2007, Kenya 2010, and Pakistan) or 2 wk (Côte d’Ivoire, Georgia, Laos, Liberia, and the Philippines). Poor sanitation was defined as unimproved sanitation and open defecation. The highest educational level was obtained for the caregiver of the child (Côte d’Ivoire, Cameroon, Kenya 2007, Kenya 2010, Laos, Nicaragua, Pakistan, and the Philippines) or head of the household (Colombia, Mexico 2006, and the United States). *^,^**Rao-Scott chi-square test: *P < 0.05, **P < 0.01. AGP, α-1-acid glycoprotein; BRINDA, Biomarkers Reflecting Inflammation and Nutritional Determinants of Anemia; CRP, C-reactive protein; NA, not available; SES, socioeconomic status.

### Anemia associated with ID

**FIGURE 1:**
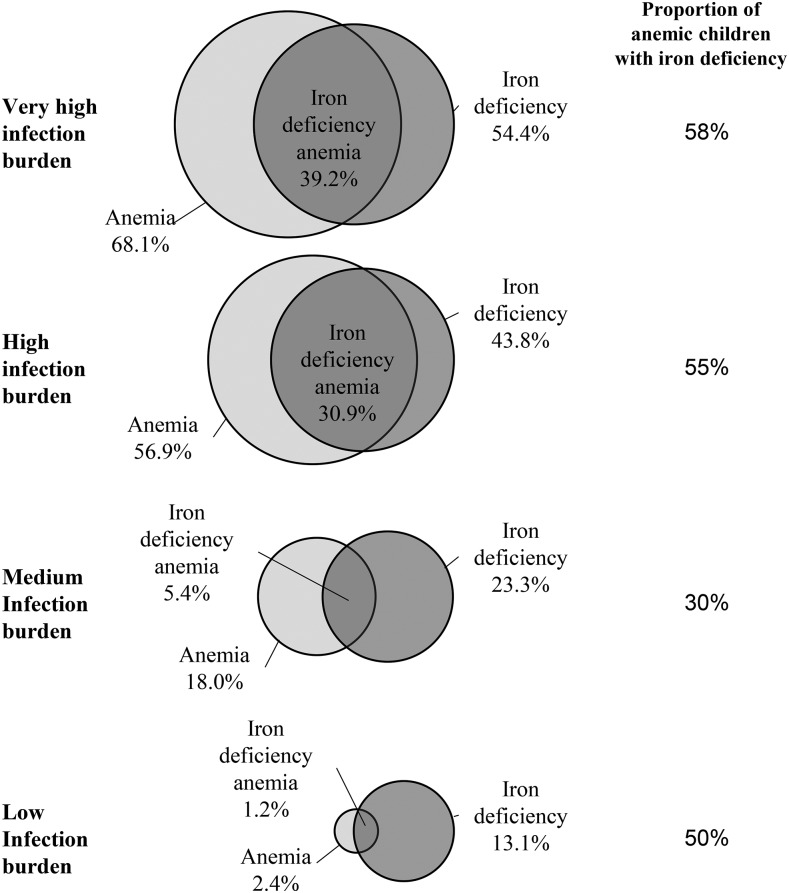
Venn diagrams illustrating the prevalence of iron deficiency, anemia, iron deficiency and anemia, and proportion of anemic individuals with iron deficiency in preschool children by category of infection burden: Biomarkers Reflecting Inflammation and Nutritional Determinants of Anemia (BRINDA) project. Values are proportions. Iron deficiency was defined as an inflammation-adjusted ferritin concentration <12 μg/L. Ferritin values were adjusted by regression with the use of reference CRP and AGP concentrations that were equivalent to the first decile of a reference population. Values were adjusted for CRP and AGP when both were available or for only CRP or AGP if only one was available. Any inflammation defined as was defined as a CRP concentration >5 mg/L or AGP concentration >1 g/L. Only AGP data were available in Nicaragua and Pakistan. Only CRP data were available in Colombia, Georgia, Mexico (2006 and 2012), and the United States. Anemia was defined as a hemoglobin concentration <110 g/L. Countries were grouped as follows—low infection burden: Georgia and the United States; moderate infection burden: Colombia, Mexico (2006 and 2012), and Nicaragua; high infection burden: Bangladesh, Laos, Pakistan, Papua New Guinea, and the Philippines; and very high infection burden: Cameroon, Côte d’Ivoire, Kenya (2007 and 2010), and Liberia. AGP, α-1-acid glycoprotein; CRP, C-reactive protein.

The relative prevalences of anemia, ID, and IDA (defined as having both anemia and ID) varied widely by infection group and among countries within infection groups (**Supplemental Figures 3** and **4**). The proportion of anemic PSC who had unadjusted ferritin concentrations <12 μg/L ranged from <1% in Georgia to 57% in the United States with an (unweighted) mean of 28% for all surveys. After ferritin concentrations were adjusted for inflammation, the proportion of anemic PSC with ID (regression-adjusted ferritin concentration <12 μg/L) increased in all surveys (unweighted mean of 43% for all surveys). In pooled analyses, the proportion of anemic PSC with ID, which was defined with the use of unadjusted ferritin concentrations, was 50%, 23%, 48%, and 26% in the low-, moderate-, high-, and very high–infection burden groups, respectively (**Supplemental Figure 5**); the corresponding values were 50%, 30%, 55%, and 58%, respectively, with the use of inflammation-adjusted ferritin concentrations (**Figure 1**). The large change in the proportion of anemic individuals with ID in the very high–infection group was explained by the number of anemic individuals with inflammation who were newly classified as iron deficient after adjustment for inflammation (∼17% of the total population) compared with a moderate increase in ID cases in nonanemic individuals (**Supplemental Figures 6–9**).

**FIGURE 2:**
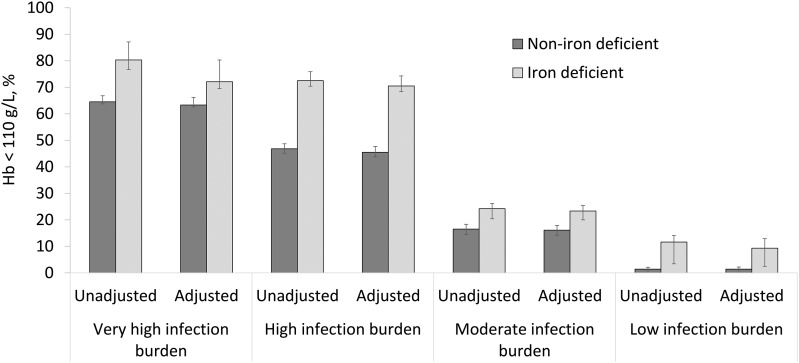
Prevalence (95% CI) of anemia in children with and without iron deficiency by category of infectious disease burden and adjustment of an iron-status indicator (ferritin) for inflammation: Biomarkers Reflecting Inflammation and Nutritional Determinants of Anemia (BRINDA) project. Ferritin values were adjusted by regression with the use of reference CRP and AGP concentrations that were equivalent to the first decile of a reference population. Values were adjusted for CRP and AGP when both were available or for only CRP or AGP if only one was available. Countries were grouped as follows—low infection burden: Georgia and the United States; moderate infection burden: Colombia, Mexico (2006 and 2012), and Nicaragua; high infection burden: Bangladesh, Laos, Pakistan, Papua New Guinea, and the Philippines; and very high infection burden: Cameroon, Côte d’Ivoire, Kenya (2007 and 2010), and Liberia. AGP, α-1-acid glycoprotein; CRP, C-reactive protein; Hb, hemoglobin.

The examination of the prevalence of anemia in individuals with and without ID provided a somewhat different picture, whereby the absolute difference in the prevalence of anemia in individuals with and without ID in the very high–infection group was 16 pp when unadjusted ferritin was used and only 9 pp when adjusted ferritin was used (**Figure 2**). The difference in anemia prevalence in individuals with and without ID (using adjusted ferritin) was 25, 7, and 8 pp in the high-, moderate-, and low-infection groups, respectively, and was minimally affected by the adjustment to ferritin (≤2.3 pp).

### Multivariable models of predictors of anemia

There was significant heterogeneity (*P* < 0.001) in relations between anemia and age, breastfeeding status, and inflammation between infection groups. Within infection groups, there was significant heterogeneity in relations between anemia and individual covariates by survey for a number of covariate–infection-group combinations although, in general, the relations were in a similar direction across surveys as suggested in Tables 2–6.

In exploratory multivariable models, child age, ID (except for severe anemia in the very high–infection group), and stunting (except for anemia in the low-infection group) independently predicted anemia in infection subgroups (**Table 7**). In addition, inflammation was associated with anemia in the high- and very high–infection groups but not in the low- and moderate-infection groups. In the very high–infection group only, male sex was independently positively associated with anemia, and malaria was associated with both anemia and severe anemia. Other independent predictors of anemia and severe anemia differed by the setting, and included wasting, household sanitation and water, VAD, and rural location. Several interactions with age remained significant in final multivariable models although the specific interactions that were detected differed by the infection group; these interactions included those between age and inflammation, sanitation, ID, and wasting.

**TABLE 7:** Logistic regression models of factors associated with anemia and severe anemia in preschool children by category of infection burden: the BRINDA project^1^

	Anemia (hemoglobin concentration <110 g/L)								Severe anemia (hemoglobin concentration <70 g/L)			
	Low infection burden		Medium infection burden		High infection burden		Very high infection burden		High infection burden		Very high infection burden	
Variable	OR (95% CI)	*P*	OR (95% CI)	*P*	OR (95% CI)	*P*	OR (95% CI)	*P*	OR (95% CI)	*P*	OR (95% CI)	*P*
Country	NA	<0.0001	NA	<0.0001	NA	<0.0001	NA	<0.0001	NA	<0.0001	NA	<0.0001
Age, mo	NA	NA	NA	NA	NA	NA	NA	NA	NA	NA	NA	NA
Sex, M	0.44 (0.16, 1.21)	0.11	0.95 (0.76, 1.21)	0.70	0.96 (0.85, 1.10)	0.60	1.35 (1.14, 1.60)	0.0006	1.08 (0.79, 1.48)	0.64	1.15 (0.81, 1.62)	0.44
Iron deficiency	6.34 (1.81, 22.24)	0.004	NA	NA	NA	NA	1.63 (1.33, 1.99)	<0.0001	NA	NA	NA	NA
Inflammation	1.59 (0.68, 3.72)	0.29	1.07 (0.79, 1.45)	0.65	NA	NA	NA	NA	0.95 (0.67, 1.34)	0.75	NA	NA
Malaria	NA	NA	NA	NA	NA	NA	3.06 (2.32, 4.05)	<0.0001	NA	NA	1.73 (1.17, 2.56)	0.006
Stunted	0.33 (0.13, 0.80)	0.014	1.84 (1.38, 2.45)	<0.0001	1.69 (1.48, 1.92)	<0.0001	1.36 (1.08, 1.71)	0.010	1.88 (1.37, 2.57)	<0.0001	2.00 (1.45, 2.75)	<0.0001
Wasted	NA	NA	NA	NA	NA	NA	NA	NA	NA	NA	1.97 (1.08, 3.58)	0.027
Unimproved sanitation	NA	NA	NA	NA	1.67 (1.21, 2.30)	0.002	NA	NA	NA	NA	NA	NA
Unimproved water source	NA	NA	NA	NA	NA	NA	NA	NA	1.73 (1.05, 2.84)	0.032	NA	NA
Vitamin A deficiency	NA	NA	NA	NA	NA	NA	NA	NA	NA	NA	2.14 (1.31, 3.47)	0.002
Rural location	NA	NA	NA	NA	NA	NA	NA	NA	NA	NA	0.54 (0.31, 0.94)	0.029
Age × inflammation												
Inflammation at 12 mo	NA	NA	NA	NA	1.71 (1.40, 2.08)	<0.0001	1.59 (1.23, 2.06)	0.0004	NA	NA	3.50 (1.79, 6.86)	0.0003
Inflammation at 48 mo	NA	NA	NA	NA	1.09 (0.87, 1.36)	0.032	2.70 (1.85, 3.95)	<0.0001	NA	NA	11.13 (3.29, 37.66)	0.0001
Age × sanitation												
Unimproved sanitation at 12 mo	NA	NA	NA	NA	NA	NA	0.83 (0.55, 1.27)	0.22	1.40 (0.87, 2.26)	0.17	NA	NA
Unimproved sanitation at 48 mo	NA	NA	NA	NA	NA	NA	1.34 (0.88, 2.03)	0.17	3.58 (1.72, 7.42)	0.0006	NA	NA
Age × iron deficiency												
Iron deficiency at 12 mo	NA	NA	2.20 (1.40, 3.47)	0.0006	3.51 (2.81, 4.38)	<0.0001	NA	NA	3.25 (2.12, 4.96)	<0.0001	0.55 (0.34, 0.87)	0.011
Iron deficiency at 48 mo	NA	NA	1.02 (0.70, 1.47)	0.94	2.21 (1.78, 2.73)	<0.0001	NA	NA	0.92 (0.53, 1.59)	0.77	2.00 (0.72, 5.56)	0.19
Age × wasting												
Wasting at 12 mo	<0.001 (<0.001, <0.001)	0.015	NA	NA	NA	NA	NA	NA	NA	NA	NA	NA
Wasting at 48 mo	0.14 (0.02, 1.04)	0.055	NA	NA	NA	NA	NA	NA	NA	NA	NA	NA

1 Countries were grouped as follows—low infection burden (*n* = 3175): Georgia and the United States; moderate infection burden (*n* = 8532): Colombia, Mexico (2006 and 2012), and Nicaragua; high infection burden (anemia and severe anemia: *n* = 9554): Bangladesh, Laos, Pakistan, and the Philippines; and very high infection burden (anemia: *n* = 4425; severe anemia: *n* = 4481): Cameroon, Côte d’Ivoire, Kenya (2007 and 2010), and Liberia. Age (as a continuous variable, in months), sex, iron deficiency, inflammation, malaria (for very high–infection countries only), and an indicator variable for the survey were forced into models. Interaction terms were evaluated by calculating ORs at 12 and 48 mo of age. For anemia models, the variables that were not significantly associated with the outcome and thus were removed from the model were as follows—very high infection: VAD, rural or urban location, and socioeconomic status; high infection: none; medium infection: wasting and rural or urban location; and low infection: stunting and wasting. For severe anemia, variables that were removed from the model were as follows—very high infection: rural or urban location, socioeconomic status, and sanitation; and high infection: none. The model development included interactions with age (see Methods for details). Anemia was defined as a hemoglobin concentration <110 g/L, and severe anemia was defined as a hemoglobin concentration <70 g/L. Iron deficiency was defined as an inflammation-adjusted ferritin concentration <12 μg/L (32). Vitamin A deficiency was defined as an inflammation-adjusted retinol concentration (Colombia, Mexico 2012, and Nicaragua) or retinol-binding protein concentration (all other surveys) <0.70 μmol/L (34). Iron and vitamin A values were adjusted by regression with the use of reference CRP and AGP concentrations that were equivalent to the first decile of a reference population. Values were adjusted for CRP and AGP when both were available or for only CRP or AGP if only one was available. Any inflammation was defined as a CRP concentration >5 mg/L or AGP concentration >1 g/L. Only AGP dates were available in Nicaragua and Pakistan, and only CRP data were available in Colombia, Georgia, Mexico (2006 and 2012), and the United States. Stunting, underweight, and wasting were defined as *z* scores <−2 SDs for height-for-age, weight-for-age, and weight-for-height or -length, respectively, with the use of WHO growth standards. AGP, α-1-acid glycoprotein; BRINDA, Biomarkers Reflecting Inflammation and Nutritional Determinants of Anemia; CRP, C-reactive protein; NA, not available.

## DISCUSSION

In this analysis of data from nearly 30,000 PSC from 16 cross-sectional surveys, we identified nutritional and nonnutritional factors that are associated with anemia and severe anemia in young children. Consistent with conceptual models of anemia (8), there were clear associations with ID, stunting, underweight, inflammation, unimproved household sanitation, and low SES. Some associations, such as ID, were consistent across all surveys. Other factors, such as inflammation, were associated with anemia primarily in settings with a greater burden of infectious disease.

These results are useful for identifying geographic areas where, and population subgroups in whom, anemia and severe anemia are likely to be prevalent. Findings from this work highlight the context-dependent nature of anemia both in terms of the total burden and the individual characteristics and environmental conditions that are associated with anemia. Specifically, in countries with a high burden of infectious disease and infectious disease risk factors, anemia prevalence was high, and anemia was associated with variables that are related to infection, sanitation, and inflammation in addition to iron status. In contrast, in settings with lower infectious disease burden, the total prevalence of anemia was lower, and ID appeared to be a more important predictor of anemia. These results are consistent with a recent analysis of NHANES data from the United States where an estimated 38% of anemia in children aged 12–59 mo was attributable to ID (35).

The positive relation between breastfeeding and anemia was no longer significant after controlling for child age in 5 of 9 surveys for which the bivariate association was significant. This association between anemia and breastfeeding in the remaining 4 countries likely reflected confounding by other individual or household characteristics (e.g., children in poor households may be more likely to be anemic and more likely to receive continued breastfeeding than are children in wealthier households), but an in-depth exploration of these pathways was beyond the scope of this paper. The association may also be explained by inadequate complementary feeding practices in this sample of children >6 mo of age, but the dietary intake data needed to confirm this explanation were limited.

Hemoglobin concentrations are commonly measured in large surveys, such as in Demographic and Health Surveys, which enable the estimation of the global burden of anemia and trends (1). However, indicators of potential causes of anemia (such as iron-status biomarkers, infections, and hemoglobinopathies) are not routinely measured in surveys to assess anemia prevalence; this lack of data limits efforts to understand the etiology of anemia and to develop appropriate interventions. A large modeling analysis, which used data and methods from the Global Burden of Diseases, Injuries, and Risk Factors 2010 study, applied estimated shifts in hemoglobin concentrations (derived from literature reviews and meta-analyses) from interventions, such as iron supplementation, to estimate the cause-specific attribution for anemia (8). The authors estimated that ID was the primary cause of anemia globally (with an average contribution close to 50%) followed by hookworm infection, genetic hemoglobin disorders, and malaria, although there was some heterogeneity in the relative importance of causes across regions (8). In a recent WHO report, the estimated proportion of “anemia that is amenable to iron supplementation” in children 6–59 mo of age in 2011, which was also estimated by applying shifts in the mean hemoglobin concentration to population hemoglobin distributions, was 32% in the Africa region, 41% in South-East Asia, and 56% in the Americas (36). Our analyses, which used individual-level data, are consistent with the emphasis on both nutritional and nonnutritional causes and the heterogeneity of anemia etiology by region.

The observed proportion of anemic individuals with concomitant ID increased after the adjustment of ferritin concentrations for inflammation, thereby suggesting that a failure to measure and adjust mathematically for indicators of inflammation may underestimate the underlying prevalence of ID and IDA (32). With the use of the inflammation-adjusted ferritin values, the proportion of anemic PSC who also had ID was ∼50% for the low-, high-, and very high–infection groups. However, the corresponding value in the moderate-infection group was only 30%; note that all surveys in this group only measured a single acute-phase protein (either CRP or AGP), and the ferritin adjustment had a relatively smaller impact on the prevalence of ID in these surveys (32). Thus, it is possible that the use of 2 acute-phase proteins to adjust ferritin concentrations would increase the estimated prevalence of ID and the proportion of anemia that is associated with ID in these countries.

We report the prevalence of IDA as it is conventionally defined (concomitant ID plus anemia) as well as the proportion of anemic individuals with ID. However, these numbers are not equivalent to the proportion of anemia that will be resolved by the provision of iron because a child with ID may also have anemia that is caused by another factor. In the BRINDA data set, in the 54% of children with ID in the group with very high infection burden (countries in sub-Saharan Africa), the prevalence of anemia was 72%, but in PSC without ID, the prevalence of anemia was 63%. In this example, theoretically eliminating ID would reduce the prevalence of anemia by a maximum of 9 pp (i.e., 72–63 pp). In the group with high infection burden (countries in Asia), the prevalence of anemia was 70.5% in PSC with ID and 45.5% in those without ID, which suggested that, hypothetically, there may be a greater impact of iron interventions in these settings. However, simply examining the proportion of anemic children with ID (58% and 55% for countries with very high and high infection burdens, respectively) obscures this distinction.

This suggestion is supported by the results of a recent trial in a population of children age 12–36 mo in Côte d'Ivoire with high IDA prevalence at baseline (24–38% IDA by group, defined as ID plus anemia) (37). In this trial, the provision of iron-fortified complementary foods (CFs) for 9 mo reduced the prevalences of ID and IDA in the CF groups compared with those in the control group at 9 mo postintervention, but reductions in total anemia prevalence were less pronounced: at 9 mo postintervention, the prevalence of IDA was 18.7%, 1.2%, and 3.4%, and total anemia prevalence was 71.1%, 70.4%, and 65.5% in the control, CF ferrous fumarate, and CF ferric pyrophosphate groups, respectively. These results suggest that defining IDA simply as having ID plus anemia, while indicative of a potential benefit of iron interventions, may overestimate the reduction in total anemia prevalence that would be expected after the provision of iron (even when issues of dosage, duration, and compliance are disregarded). We did not present population-attributable risk calculations because they are considered inappropriate for cross-sectional data from which causality cannot be inferred; however, because of the known biological relations between ID and anemia, future efforts to explore the application of population-attributable risk estimates may be useful for studies that aim to predict the proportion of anemia that could be resolved through iron-intervention programs.

A second caveat with regard to programmatic implications of delivering iron is that uncertainties remain about how best to manage iron-intervention programs in the context of a high burden of infectious disease. The administration of iron during inflammation may be ineffective if hepcidin is elevated (38) and may also be harmful in the case of certain infections (12, 13). The regression adjustment for ferritin used in this article and described by Namaste et al. (7) uses lower reference values for markers of inflammation in comparison to previous methods. Thus, presumably, this method identifies a greater proportion of cases of underlying iron deficiency which may be masked by low-level inflammation. Thus, this method may be useful to better estimate the underlying prevalence of ID and to monitor trends and responses to intervention, although longitudinal studies are needed to confirm the validity of this approach. However, decisions about when, how, and to whom to administer iron should take into account potential interactions with infections.

A major strength of this analysis is the availability of individual-level data on potential risk factors for anemia from 16 surveys from diverse geographic regions yielding large pooled sample sizes for groups with varying burden of infections. However, missing information on other potential causes of anemia (e.g., genetic factors, helminths and other infections, and B vitamin status) still limits the extent to which we could examine all possible contributors to anemia. In addition, several methodologic factors affect the measurement of anemia. The use of capillary blood samples (as in the majority of surveys included here) can potentially underestimate hemoglobin concentrations if excessive pressure is applied to the finger during blood collection. Differences in laboratory methods could also introduce error into the results although this potential source of variation did not appear to affect our ability to detect patterns of association with anemia across surveys. Finally, an important limitation of the use of cross-sectional data is the inability to make inferences about the etiology of anemia. Nevertheless, the bivariate associations are useful for identifying individuals or population subgroups who are likely to be anemic.

The identification of independent relations between anemia and biomarkers of infection, inflammation, and micronutrient status is complicated by the biological interactions between infection and nutritional status. With current methods, an individual’s underlying iron or vitamin A status (stores) cannot be known with certainty when inflammation is present. We have addressed this issue, to the extent possible, by using what we believe are novel methods to adjust markers of ID and VAD for the presence of systemic inflammation that do not rely on arbitrary cutoffs for CRP and AGP, although further evidence for the validity of these approaches is needed.

We used a hemoglobin-concentration cutoff of 110 g/L to define anemia for consistency with WHO guidelines for children aged 6–59 mo (29). However, there has been some evidence to suggest that this value could overestimate the prevalence of anemia, particularly for younger children, if the statistical cutoff of a mean of –2 SDs (although not necessarily a gold standard) is applied to estimate the threshold for anemia. NHANES II data were used to support the WHO 2001 anemia cutoffs, but in those data, the concentration that was equivalent to the mean of –2 SDs of hemoglobin in non-ID children was 105 g/L for children aged 0.5–0.99 y, and 107 g/L for children aged 1.0–4.99 y (29, 39). In addition, concentration cutoffs of 105 g/L at 4–6 mo of age and 100 g/L at 9 mo of age were suggested on the basis of data from iron-replete infants in a supplementation study (40). We observed positive relations between age and hemoglobin in all but 3 surveys, and the associations between age and anemia were independent of other risk factors for anemia as have been observed elsewhere (39, 41). By controlling for age in the multivariable models, we were able to examine the independent associations of other characteristics with anemia.

In exploratory multipredictor models, we observed interactions with age and selected covariates. In general, these interactions suggest that ID may be a more important predictor of anemia in younger children and that inflammation and related variables (such as household sanitation) are more important in older children (all age ≤59 mo in this analysis), thereby implying that anemia-control efforts may be strengthened by age-specific actions. These relations should be further examined with the use of appropriate analytic methods such as a path analysis.

Despite efforts to implement programs to address child anemia, there has been limited progress toward this objective globally and particularly in sub-Saharan Africa. In general, our results suggest that, in settings with low and moderate burdens of infectious disease, nutritional factors such as ID are important contributors to anemia. In contrast, in settings with a high burden of infectious disease, the contributors to anemia are more diverse, and both nutrition and infectious disease control are important factors to address.

In conclusion, the heterogeneity in results by country indicates that data collection with a focus on potential risk factors for anemia should be a priority to understand the likely etiology of anemia in a setting before designing programs. Indeed, risk factors for anemia may also differ within countries where there is diversity in the geography, dietary patterns, and burden of infectious disease. In addition, data on nonmodifiable risk factors for anemia (such as hemoglobinopathies) are needed to assess the extent to which anemia-control programs can be expected to have an impact. Concerns have been raised about possible risks of providing additional iron to individuals with adequate iron status and/or to individuals who are living in settings with high burdens of malaria and other infectious diseases (42). Until a rapid, inexpensive diagnosis of ID and inflammation in individuals is feasible, estimates of the prevalence of ID and IDA in a country or geographic region can provide guidance to programs. Where feasible, iron-status indicators, such as ferritin, should be measured in nutrition surveys, and indicators of inflammation, such as CRP and AGP, should be measured concurrently, to permit the interpretation of ferritin concentrations. Finally, the burden and distribution of infectious disease should be considered in decisions regarding when, where, and to whom to provide iron. Interactions with age should be considered for relevance to policy decisions including program targeting.
